# Death from Mercurial Fumigation

**Published:** 1840-01

**Authors:** 


### DEATH FROM THE INHALATION OF THE VAPOUR OF THE BI-SULPHURET OF MERCURY.

A patient in the surgical ward of the Louisville Hospital, labouring under a venereal ulcer of the palate, was directed to use mercurial fumigation, which he did, in the manner and to the degree, usually practised in the ward, but it proved fatal to him in less than half an hour. A detailed report of the case with the *post mortem* appearances will be given in our next number. D.

